# Energy Expenditure, Availability, and Dietary Intake Assessment in Competitive Female Dragon Boat Athletes

**DOI:** 10.3390/sports5020045

**Published:** 2017-06-21

**Authors:** Jun Liang Ong, Iain A. Brownlee

**Affiliations:** Human Nutrition Research Centre, Newcastle University, 172A Ang Mo Kio Ave 8, Singapore 567739, Singapore; junliang.ong@gmail.com

**Keywords:** female athletic triad, energy expenditure, energy availability, dietary intake

## Abstract

Dragon boat racing requires high physical activity levels during competition and training. The female athletic triad refers to a number of negative health consequences (e.g., amenorrhoea, low bone mineral density, and low energy availability) that may result from high physical activity in female athletes in parallel with inadequate dietary intake. This study aimed to estimate energy expenditure and dietary adequacy in female competitive dragon boat athletes. Following ethical approval, energy expenditure was assessed by use of Sensewear^TM^ armbands (which measure movement as well as galvanic heat loss) on nine dragon boat athletes preparing for the Southeast Asian Games 2013. The mean estimated energy expenditure for the athletes was 2226 ± 711 kJ/day. Mean total energy, recorded using three-day food diaries (6715 ± 2518 kJ/day) and energy availability (99 ± 56 kJ/kg/day), were low. Estimated micronutrient intake (calcium 699.3 ± 328.7 mg/day and iron 10.6 ± 4.7 mg/day) did not meet recommended daily allowances of 800 mg/day and 19 mg/day, respectively. The low intake of energy, calcium, and iron noted within this study could have negative effects on performance and short- and long-term health in female dragon boat athletes.

## 1. General Introduction

Dragon boat racing is an international water sport originating from China, with competitive and recreational teams typically comprised of 20 paddlers, a drummer, and a steersperson in a carbon-fiber boat [1]. Race distance ranges from 200 to 2000 m [2]. Race results obtained from the International Dragon Boat Federation [3] suggests that 1000 m races are completed in less than four minutes on average. Dragon boating is a full body, high intensity exercise in which the athletes propel the boat forward with a short paddle with a horizontal cross piece at the top end. The paddle is held firmly in both hands by the cross piece and on the shaft close to the blade. Dragon boat racing represents a highly vigorous form of competitive physical activity. Throughout China, Southeast Asia and beyond, schools, community clubs, and private organizations are forming teams to compete against each other in various competitions held at national and international levels. Training schedules can include high amounts of physical activity per day, particularly in the run-up to major competitions [2].

The female athletic triad is a term describing the dietary inadequacy of female athletes in relation to their high energy expenditure and nutrient requirements. Within the female athletic triad, an interrelationship of menstrual disorder, low energy availability, and decreased bone mineral density [4] exists which threatens the health of women with high physical activity [5]. Alongside these issues, it has been suggested that athletes with low energy availability may be at greater risk of upper respiratory tract infections [6]. Falling ill when preparing for competition could affect performance due to fatigue and might reduce training efficiency and increase absenteeism, with subsequent impacts on teamwork and competitive performance. The potential for negative health and performance outcomes is therefore likely to be exacerbated by inadequate dietary intakes of energy, with other health issues resulting from inadequate intake of calcium and iron [7].

Energy availability is the difference between energy intake and energy expended during exercise (relative to fat-free mass) and represents the energy remaining for other metabolic processes to ensure proper physiological functions. Energy balance is the difference between energy intake and total energy expenditure; hence, it is an output of energy in which body physiological functions could be suppressed in severe cases of low energy availability. Therefore, energy availability is a better estimation for the adequacy of energy intake as compared to energy balance [6]. Low energy availability of <125.5 kJ/kg body fat-free mass/day, which is a threshold near resting metabolic rate [8], is associated with the female athlete triad [9].

Accurately assessing energy expenditure in athletes is challenging due to the need for specialized equipment that does not interfere with their ability to perform during training or competition. Armband-based technology has been suggested to be a relevant approach to measure energy expenditure in athletes, because it is non-invasive and interferes minimally with sporting activities [10].

There is currently a lack of data on energy expenditure and dietary adequacy in female dragon boat athletes (paddlers). Therefore, the objectives of this study were to investigate the energy expenditure in elite, female, Singaporean dragon boat athletes using armband devices and to estimate their energy availability, calcium and iron intake using three-day food diaries.

## 2. Methods

### 2.1. Participants

Ethical approval for this study was obtained from Newcastle University Faculty of Science, Agriculture and Engineering Ethics Committee (ID: 13-BRO-052, approval granted on 24 January 2014) in accordance to the Declaration of Helsinki. Following informed consent, a total of 11 female elite athletes were recruited. Two subjects dropped out during the course of study due to discomfort caused by the armband. The final study sample consisted of nine athletes who completed the study over a course of 14 days whilst training for the South East Asian (SEA) Games 2013.

### 2.2. Data Collection

A Sensewear^TM^ armband (BodyMedia, Pittsburgh, PA, USA) was used to assess energy expenditure. This armband continuously measures triaxial accelerometry and also has a heat sensor to assess galvanic sensor that rests against the skin and estimates bodily heat loss [11]. All participants were briefed on the usage of Sensewear^TM^ armbands and instructed on how to complete their food diary. Participants were also given the opportunity to try on the armband and ask additional questions. The armbands were worn on the ‘top arm’ (i.e., the arm that holds the horizontal handle of the paddle) to minimize contact with water.

Before commencement of training, the armbands were strapped to the triceps as this is suggested to be the best location in terms of wearability and utility of sensors [12]. The armbands were positioned to ensure comfort and to avoid over-tightening of the Velcro straps. All armband devices were fully charged and disinfected in between measurements. A total of two measurements were taken due to the limited number of armbands. Five participants had measurements taken on three weekdays (Monday, Tuesday, and Thursday) and four on two weekends and one weekday (Saturday, Sunday, and Monday). Data were extracted using software Sensewear Professional 7.0 version 7.0.0.2378.

Athletes were requested to complete a three-day food diary in conjunction with the assessment of energy expenditure. This approach to estimate dietary intake is sufficient to analyze habitual food intake and places less participant burden on participants than prolonged data collection [13]. Portion size was estimated using household measures. All recorded data were processed using the dietary software program WinDiets 2010 (Robert Gordon, University of Aberdeen, Aberdeen, UK) in order to estimate the dietary intake of energy, calcium, and iron. Energy and nutrient composition of local foods was obtained from the “Energy and Nutrient Composition of Foods” database produced by Singapore’s Health Promotion Board [14].

Reported dietary intake was considered against basal metabolic rate (BMR) estimates. A cut-off of energy intake:BMR (EI:BMR) < 1.75 was used to classify ‘under-reporters’ for physically active individuals and ≥2.4 for ‘over-reporting’ behavior as previously described [15]. BMR was calculated using a previously described methodology (BMR (kJ/day) = 4.184 × (669 + 13 (body mass in kg) + 192)) for that has been used for Southeast Asian athletes [16].

### 2.3. Assessment of Body Composition

Body composition was measured at the end of the dietary data collection period. Body mass (to the nearest 0.1 kg) and body fat percentage were measured using a portable bioelectrical impedance monitor (Karada Scan, model HBF-362, Omron, Osaka, Japan). Participants were dressed in light clothing and bare-footed when the measurement was taken. Participants provided a self-reported estimate of height.

### 2.4. Data Analysis

The energy availability of all participants was calculated as previously described [6]. Specifically, the following equation was used: Energy availability = (Energy intake (kJ) − Energy expenditure during exercise (kJ))/fat-free mass (kg). Descriptive statistics (mean and SD) were conducted on body compositions, dietary intake, and energy status. Statistical analysis was completed using Minitab^®^ 16.2.4, with *P* < 0.05 considered as statistically significant. All data were noted to be normally distributed (*P* > 0.05).

A one sample *t*-test was conducted to compare the difference of energy availability with reference values previously associated with increased risk of menstrual disorder, infertility, decreased bone mineral density and upper respiratory tract infection (<125.5 kJ/kg∙fat-free mass (FFM)/day), or normal physiological functions/improved sporting performance (≥188 kJ/kg∙FFM/day) [6]. Dietary calcium and iron intake data were also compared with recommended dietary allowance targets of 800 mg/day and 19 mg/day respectively [17] using one-sample *t*-tests to consider the adequacy of intake. Linear regression analysis and Pearson’s correlation analysis were conducted to investigate the relationship between various anthropometric measurements and energy expenditure, as well as dietary energy, calcium, and iron to energy intake.

## 3. Results

Participants ranged in age from 21 to 26 years and had a mean body mass of 59.8 ± 8.4 kg (see Table 1). The mean total energy expenditure was 8083 ± 805 kJ/d. The correlation coefficient of body mass, body fat mass, fat-free mass, and BMI in relation to the amount of energy expended during dragon boat training did not suggest a significant association (*P*-values of 0.196, 0.234, 0.217, and 0.390 respectively).

Analysis of energy status and dietary intake are summarized in Table 2 below. From these data, all nine participants had EI:BMR ratio <1.75 with a mean ± SD of 1.10 ± 0.39, suggesting that dietary records were under-reported. The mean was skewed towards zero, indicating that the majority of reported energy intakes were extremely low. Only two of the nine participants had EI:BMR ratio close to 1.75. Five of the nine participants had reported energy intake lower than their estimated BMR. The mean percentage energy from carbohydrates, proteins and fat were 41.2 ± 12.2, 24.7 ± 8.3, and 34.2 ± 13.7%, respectively. Eight out of the nine participants had estimated energy availability less than 188 kJ/kg FFM/day, with six having values much lower than 125.5 kJ/kg∙FFM/day. The mean ± SD estimated energy availability of 99.2 ± 55.6 kJ/kg/day was also lower than this value but was not significantly different (*P* = 0.193).

Mean dietary calcium (699 ± 329 mg/day) and iron intake (10.6 ± 4.7 mg/day) were below the recommended dietary allowance of 800 mg and 19 mg respectively. Mean dietary iron consumption was statistically lower than these recommended values (*P* < 0.001) with eight out of nine participants having insufficient intake.

All participants completed standard training regimes consisting of approximately 1 h of weights (resistance training) and 5 km jog followed by 2 h of rowing, twice a day for a month. A total of six days of team training activities were recorded in this study consisting of four weekdays and two weekends. That accounts to a mean estimated physical activity energy expenditure of 2225.9 ± 709.2 kJ/day. BMR and physical activity during training were similar amongst the participant but the energy intake was considerably more variable.

## 4. Discussion

To the authors’ knowledge, this is the first study that has estimated energy expenditure and dietary intake in this population (female dragon boat athletes). Nine participants completed the study with the use of the armbands, with an estimated average physical activity energy expenditure of around 2226 kJ/day. The training regime undertaken would be expected to equate to a much higher energy expenditure and this estimate appears to be low in comparison to other sports with similar intensities and training regimes. Rowing expends 4184 to 8368 kJ per 1–2 h training depending on intensity [18]. Hill and Davies [19] reported total energy expenditure of 16,556 kJ/day in elite lightweight female rowers using doubly-labelled water methods. Another study conducted by Hoffman, et al. [20] on recreational kayaking measured energy expenditure of 1418 kJ in 19.5 min using heart rate monitors. The amount of estimated energy expended during the 3 h or more per day of training measured in this study was therefore much lower than those from other studies on aquatic racing sports with similar intensities.

Mean energy availability was 20% below an amount (<125.5 kJ/kg/d) that is considered to be detrimental to health. Individuals whose calculated energy intake is lower than 1.1 times their BMI are often considered ‘under-reporters’ [21]. The authors believe that this low intake of energy during competition preparation is common practice among dragon boat athletes and does not necessarily represent omissions from or misrepresentation of dietary records. Low energy intake during this training period may help athletes lose weight for the competition but there is no direct evidence to support that this approach will benefit performance. Restricted energy intake may also have more immediate effects both on risk of fatigue and injury, reduced immunity and potential infection, and overall performance during training [22]. Insufficient energy intake appears to be the major cause of low energy availability in this study. Hence, dragon boat athletes (particularly paddlers compared to drummers and steerspersons) are advised to increase their energy intake, particularly carbohydrate as the source of energy as it enhances performance and prolongs time to exhaustion [23]. Athletes should also consider carbohydrate sources that would benefit the maintenance of glycogen reserves during training and competition [24].

The individuals that had the highest energy intake also consumed more calcium and iron. This observation agrees with evidence from other groups of female athletes with intensive training regimes, where dietary (energy) restriction tends to also limit the intake of other essential nutrients [7,8]. The mean intake of calcium and iron was below recommended levels by around 13% and 44%, respectively. Non-anemic iron deficiency is consistently observed in trained female athletes due to high iron losses and inadequate dietary iron intake [9] which was consistent with the findings in this study. As both calcium and iron losses from the body tend to be relatively low, a habitual (i.e., outside of the pre-competition routine) dietary intake that included adequate intake of calcium and iron may negate short-term reduction in pre-competition intake. Low intake of iron and calcium could also lead to amenorrhea and low bone mineral density even if EA was optimal. Habitual dietary intake could also be assessed within this athlete group to better consider whether low intake of calcium and iron during training could be an issue. Future studies in this area would have to consider markers of iron and calcium/bone health status to better assess adequacy of dietary intake over longer periods of time. Long term monitoring of bone mass by dual-energy X-ray absorptiometry and/or urinalysis for bone biomarkers such as telopeptides and pyridinolines that reflect bone resorption [25] may help to assess the bone health of female athletes. In the meantime, female dragon boat athletes should be encouraged to consume adequate amounts of calcium and iron within their diet, particularly during periods of intensive training.

Our results suggest that the Sensewear^TM^ armbands used here underestimated energy expenditure during training. The Sensewear^TM^ armband calculates energy expenditure based on motion and heat flux but any movement (e.g., torso rotation) that does not lead to a displacement of the arm that the device is attached to would result in little or no change in acceleration detected and could lead to an underestimation of energy expenditure. Previous studies have suggested that this device may underestimate energy expenditure for activities that do not involve arm movement such as seated activities [26] and may not provide accurate estimation of energy expenditure at very high intensity levels [27,28]. Future studies targeting longer-term and more accurate estimation of energy expenditure in this athlete group should consider using doubly-labelled water methods [19].

Although mean dietary records suggest dietary intake appears to be inadequate (see Table 2), care should be taken when considering the appropriateness of these data, as information was collected over a short period of time. Inaccurate report of food intake can lead to misrepresentation of dietary intake [29], including energy intake and micronutrients. Previous studies have suggested that the most common reasons for athletes to under-report are (1) failure to record portion size of food correctly; (2) restricting or omitting food intake during the study to ease recording process; (3) reporting food intake inaccurately to improve the perception of a healthy athlete’s diet [21,30]. All of these factors could lead to an underestimate of energy and nutrient intake. As food portion sizes were estimated here, it seems prudent that future studies should include weighed food diaries. Future studies in the area will need to carefully weigh the importance of dietary data accuracy with the potential for additional participant burden [29].

It appears as though energy expenditure during vigorous physical activity will have been underestimated to a greater degree than dietary intake. The cut-off values used within this study to estimate adequacy of calcium and iron intake are for the general population. Additional losses of these minerals may occur in athletes [4] and have not been factored into these cut-offs. If estimates of energy expenditure are low, this would lead to an over-estimation of energy availability and thereby could also have reduced the potential to measure dietary inadequacy.

## 5. Conclusions

Energy expenditure and assessment of dietary adequacy in competitive female dragon boat athletes has not, to the authors’ knowledge, been previously assessed. These findings, although preliminary, suggest that dietary intake is inadequate to meet the athletes’ nutritional requirements.

## Figures and Tables

**Table 1:** Participants’ ages and anthropometric measures.

Participant No.	Age (years)	Body Mass (kg)	Height (m)	BFM (kg)	FFM (kg)	BMI (kg/m^2^)
1	26	64.5	1.65	16.6	49.3	23.7
2	26	64.0	1.65	17.8	46.2	23.5
3	21	59.0	1.60	15.5	43.5	23.1
4	21	72.0	1.65	21.5	50.5	26.5
5	22	61.0	1.75	13.1	47.9	19.9
6	23	56.0	1.60	12.0	44.1	21.9
7	22	51.5	1.50	11.3	40.2	22.9
8	23	44.0	1.63	5.5	38.5	16.6
9	23	66.0	1.60	19.9	43.6	25.8
Mean	23	59.8	1.63	14.8	44.9	22.6
SD	1.9	8.4	0.06	4.9	4.0	3.0

Body mass was measured to the nearest 0.1 kg; BFM = body fat mass; FFM = fat free mass; BMI = body mass index; SD = Standard deviation.

**Table 2:** Energy expenditure and dietary intake data.

ID	BMR (kJ/d)	EI (kJ/d)	EI:BMR ^a^	PAEE (kJ/d)	EA ^b^ (kJ/kg/d)	Ca Intake ^c^ (mg/d)	Fe Intake ^c^ (mg/d)
1	6309.5	6071.0	0.96	1585.7	93.7	752	10.2
2	6280.2	7430.8	1.18	2138.0	114.6	621	8.5
3	6008.2	9891.0	1.65	3100.3	156.1	1174	14.6
4	6715.3	5451.8	0.81	3485.3	38.9	643	5.8
5	6117.0	6050.1	0.99	2732.2	69.5	706	10.6
6	5845.0	7125.4	1.22	1610.8	125.1	645	6.2
7	5602.4	3610.8	0.64	1987.4	40.2	356	6.6
8	5192.3	3723.8	0.72	1640.1	54.0	195	12.8
9	6389.0	11,062.5	1.73	1757.3	202.1	1202	20.1
Mean	6050.1	6715.3	1.10 *	2225.9	99.2	699	10.6 *
SD	456.1	2518.8	0.39	709.2	55.6	329	4.7

^a^ EI:BMR values of <1.75 and ≥2.4 denotes classic ‘under-reporters’ and ‘over-reporters’ respectively; ^b^ <125.5 kJ/kg/d is suggested to be detrimental to health, ≥188 kJ/kg/day is suggested to maintain normal physiological functions; ^c^ recommended dietary allowances for calcium and iron = 800 mg/day and 19 mg/day respectively; * denotes mean value significantly different (*P* < 0.05) from hypothetical comparator values; BMR = basal metabolic rate; EI = energy intake; SD = Standard Deviation, PAEE = physical activity energy expenditure; EA = energy availability.
